# Evaluating maternal death surveillance and response system in Sunyani Municipality of Bono region in Ghana from 2017-2021

**DOI:** 10.1186/s12913-024-12023-7

**Published:** 2024-12-18

**Authors:** Amara S. Ngegbai, George Khumalo Kuma, Charles L. Noora, Ernest Tei Maya, Chris Guure

**Affiliations:** 1 Department of Epidemiology and Disease Control, School of Public Health, University of Ghana, Legon-Accra, Ghana; 2 District Health Management Team-Bo, Ministry of Health and Sanitation, Gbaima Road, Bo, Sierra Leone; 3 Ghana Field Epidemiology and Laboratory Training Program, School of Public Health, University of Ghana, Legon-Accra, Ghana; 4 Sunyani Teaching Hospital & Bono Regional Health Directorate, P. O. Box: 27 Sunyani, Ghana; 5 Department of Population, Family and Reproductive Health, School of Public Health, University of Ghana, Legon-Accra, Ghana; 6 Department of Biostatistics, School of Public Health, University of Ghana, Legon-Accra, Ghana

**Keywords:** Maternal mortality, Death audits, Data quality, Data reviews, Bono region

## Abstract

**Introduction:**

Maternal death rates in Ghana have decreased overall but remain high in rural areas. The Maternal Death Surveillance and Response System (MDSR) aims to eliminate preventable maternal deaths effectively. However, its effectiveness is less pronounced at district and subdistrict levels than at national and regional levels. Despite the requirement for periodic evaluation, there is a lack of evidence supporting these assessments. This study focuses on evaluating the MDSR in Sunyani Municipal.

**Methods:**

The evaluation was conducted using a cross-sectional design, using both qualitative and quantitative approaches. Data from the District Health Information Management Systems (DHIMS) and the maternal death line list from January 2017 to December 2021 on maternal deaths were reviewed. Stakeholders, including health professionals, were interviewed. The means, proportions, and other statistical measures were calculated using Epi Info Version 7. Qualitative data underwent content analysis, and the results were visually presented in tables and flowcharts to illustrate the flow of surveillance information.

**Results:**

Most respondents were female, constituting 51.4% (19/37), with midwives comprising the largest group at 32.4% (12/37). Approximately 75.7% (28/37) were able to explain how the surveillance data has been used for public health action. The notification and reporting process was clear to 85% (17/20) of respondents. Although 94.6% (35/37) expressed willingness to notify and participate in audits at facility and community levels, the audits remained facility-based. All facilities consistently reported maternal deaths, but data storage equipment was lacking. Approximately 80% (20/25) of forms were reported within 24 h, and 88% (22/25) were investigated within seven days. Only 68% (17/25) of forms were filled out correctly, with no municipal and facility-level line list. All reported deaths came from healthcare facilities, with no community-reported maternal deaths.

**Conclusions:**

The MDSR system in Sunyani Municipal demonstrates high awareness and willingness to participate among healthcare providers but faces challenges in data accuracy and community engagement. Death audits remain facility-based, and the lack of community-reported maternal deaths and municipal-level line lists indicates gaps in comprehensive reporting and data management. To improve the system, it is recommended that community case searches be enhanced for better reporting and to address data management issues by ensuring proper data quality assessment.

**Supplementary Information:**

The online version contains supplementary material available at 10.1186/s12913-024-12023-7.

## Introduction

Globally, in 2020, maternal mortality ratio was estimated at 223/100,000 live births [1]. This translates to almost 855 women dying daily from complications of pregnancy and childbirth. Most of these deaths are in developing countries where mortality rates are excessively high with more than 500,000 women dying each year from complications of pregnancy and childbirth [1]. Approximately half of the maternal deaths in developing countries occur in sub-Saharan Africa, where the lifetime risk of maternal death is around 1 in 37, compared to 1 in 7,800 in developed countries [2].

The maternal mortality ratio in Ghana dropped from 760 per 100,000 live births in 1990 to 319 per 100,000 live births in 2015 [3]. Since 2015, the decline rate in Ghana has stagnated with the maternal mortality ratio at 308/100,000 in 2022 [4].

The Maternal Death Surveillance and Response (MDSR) is an approach that enables comprehensive data collection for decision-making and action and improving the quality of care of pregnant mothers [5]. It aims to further reduce preventable maternal mortality by including all stakeholders in the process of recognizing maternal deaths, understanding why they occur, and taking steps to prevent similar deaths. The MDSR Strategy was adopted in 2014 as part of the integrated disease surveillance and response in the country.

Ghana had a structurally sound Maternal Death Review (MDR) system before the introduction of the Maternal Death Surveillance and Response (MDSR) system which was first piloted in 2014. Ghana was amongst the few countries that were part of that pilot. The transition from Maternal Death Review (MDR) to Maternal Death Surveillance and Response (MDSR) in Ghana was driven by the need for a continuous, systematic approach to not only review maternal deaths but also ensure timely responses to prevent future occurrences. The MDSR strategy involves key steps such as the identification and notification of maternal deaths, comprehensive reviews, data analysis, responsive interventions, and continuous monitoring and evaluation. Key personnel include healthcare workers, data collectors, community health workers, and local health authorities, all supported by death review committees at various levels [5].

In Ghana, national guidelines, capacity-building initiatives, community involvement, and intersectoral collaboration are crucial for the effective implementation of MDSR. The implementation of the MDSR in Ghana is generally challenged by logistical issues, data quality, and the need for robust training and resources to ensure its effectiveness [6]. This study aimed to look at the implementation of MDSR, especially at a district level where the effect of these challenges tends to be more pronounced. Additionally, a well-established surveillance system necessitates regular evaluation to ascertain its continued relevance for the purpose for which it was initially established [7]. We evaluated the MDSR in Sunyani East Municipality, a district in the Bono region in Ghana, which is identified as one of the regions with a rising trend of maternal deaths according to the data from District Health Information Systems (DHIMS). This evaluation aimed to assess the system’s usefulness, its surveillance attributes, and its effectiveness in meeting its objectives. Therefore, understanding the performance of the MDSR system in this region is critical to inform and enhance interventions aimed at reducing maternal mortality.

## Methods

### Evaluation design

We conducted a descriptive cross-sectional study to evaluate the MDSR system in the Sunyani East Municipality between 2017 and 2021. The updated Centers for Disease Control and Prevention (CDC) guideline for evaluating public health systems (2001) [8] was used as a guide for evaluating the system.

The Updated Guidelines for Evaluating Public Health Surveillance Systems by the CDC (2001) provide a structured framework to ensure surveillance systems effectively monitor public health events and support decision-making. The guidelines emphasize a detailed description of the system’s purpose and its usefulness, objectives, and operations, including data collection, analysis, and dissemination processes. They focus on evaluating system performance attributes such as simplicity, flexibility, data quality, acceptability, sensitivity, predictive value positive, representativeness, timeliness, and stability. Additionally, the guidelines highlight the importance of assessing resource use, including personnel, funding, and technology, to determine efficiency and sustainability. Recommendations are made based on these evaluations to improve system performance and address weaknesses, ensuring robust public health infrastructure and effective responses to health threats.

### The usefulness of the surveillance system

According to the updated CDC guidelines for the evaluation of surveillance systems, a public health surveillance system is useful if it contributes to the prevention and control of adverse health-related events. Respondents were asked to describe the trend of maternal mortality and explain what the data collected on maternal deaths was used for, and what public health actions were taken based on the surveillance data. Audit reports were reviewed and asked to produce evidence on the recommendations from the reports.

### Assessment of the usefulness of the surveillance system 

Respondents were asked to describe the trend of maternal mortality, explain what the data collected on maternal deaths was used for, and what public health actions were taken based on the surveillance data. Audit reports were reviewed and nurses, surveillance officers, and other stakeholders were asked to produce evidence on the recommendations from the reports.

### System attributes

An attribute is a characteristic used to assess a surveillance system. It represents an outcome variable obtained by analyzing a set of related variables that reflect a specific aspect of the surveillance system. The definitions used for all the attributes assessed are from the CDC’s updated guidelines on the evaluation of surveillance systems as shown in the table below:

### Assessment of the system’s attributes

**Table:** 

Attribute	Definition	Assessment
**Simplicity**	CDC defines the Simplicity of a Public Health Surveillance System as both its structure and the ease of operation while still meeting its objectives	1. Are data collection tools clear, precise, and easy to use? 2. Are the reporting channels known and clear? 3. Is there a need for further training on MDSR?
**Flexibility**	CDC defines flexibility as the ability of the system to adapt to changing information needs and operating conditions with minimal additional costs.	1. Has any change occurred in the case definition, reporting system or funding source? 2. Are more personnel required for the MDSR process? 3. Is the reporting system integrated with the DHIMs and IDSR platforms?
**Acceptability**	Acceptability is the willingness of persons and organizations to participate in a surveillance system.	1. Are health workers willing to participate in the MDSR 2. Are community members willing to participate in maternal death investigation.
**Stability**	Stability is the **reliability** (ability to collect, manage and provide data properly without failure) and **availability** (ability to be operational when it is needed) of the surveillance system.	1. Are there consistency in reporting, the data 2. Is the data readily available for planning and other purposes, 3. Are there communication equipment and other material resources needed for the surveillance system.
**Timeliness**	Timeliness refers to the speed at which data is transmitted between different levels in the surveillance system.	1. Are the MDNF completed and sent to the MHD within 24 h? 2. Are MD audits done within seven days of the maternal deaths as stipulated?
**Data quality**	Is the review of processes involving clarity, quality of trainings and supervision of personnel in the surveillance system data.	1. MDF was reviewed to check for completeness of the notification forms. 2. What are the qualifications of the people who conduct maternal death audits
**Representativeness**	Representativeness is the extent to which the system accurately describes the occurrence of the disease or event over time and its distribution in the population by person and place.	1. Is the data reported by age, communities 2. Are key populations included?

### Evaluation site

Sunyani Municipal is one of the oldest districts in Bono Region. It is part of the twenty-seven administrative districts in the Bono Region of Ghana.

**Fig. 1:**
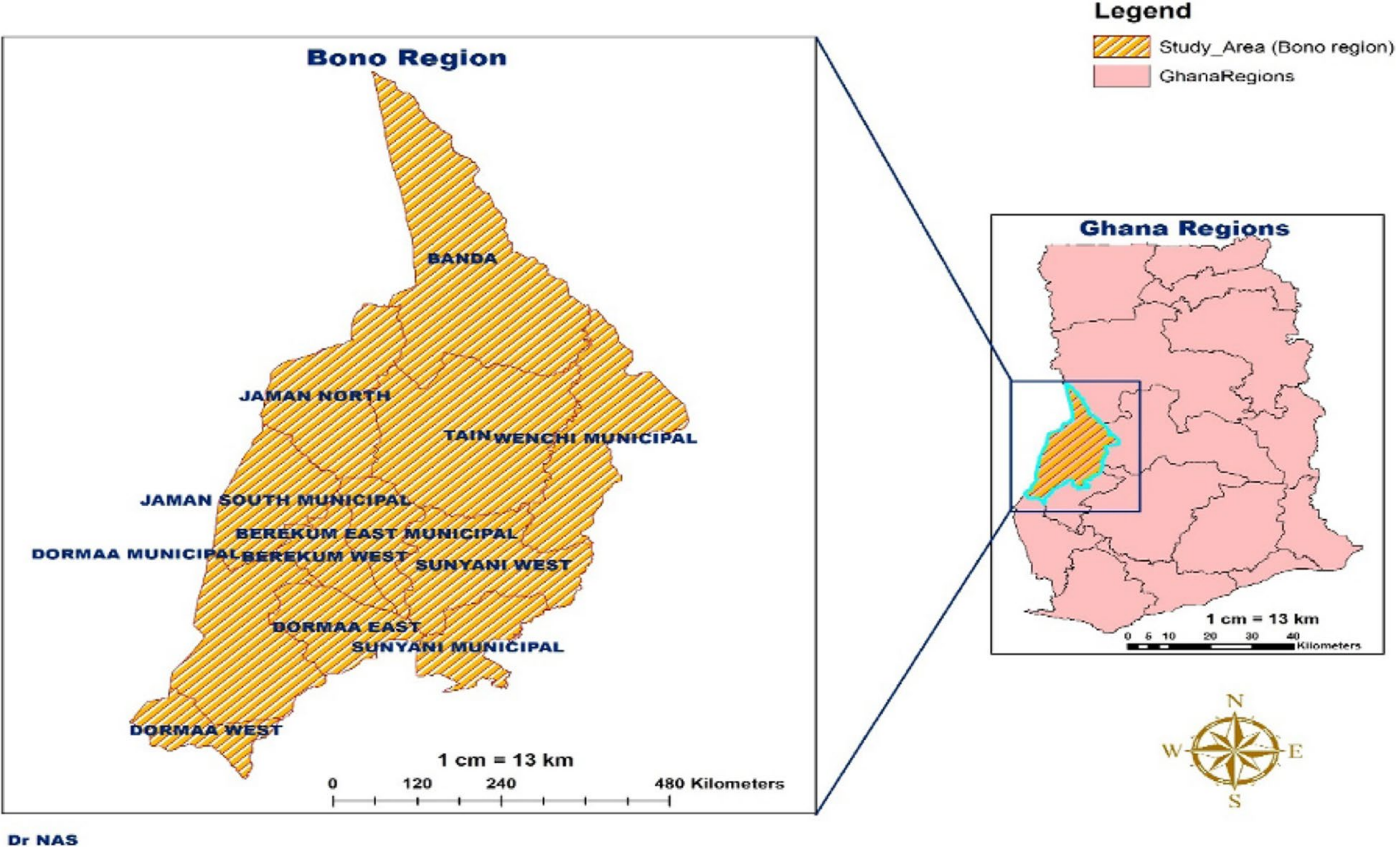
Map of Ghana showing Bono region

**Fig. 2:**
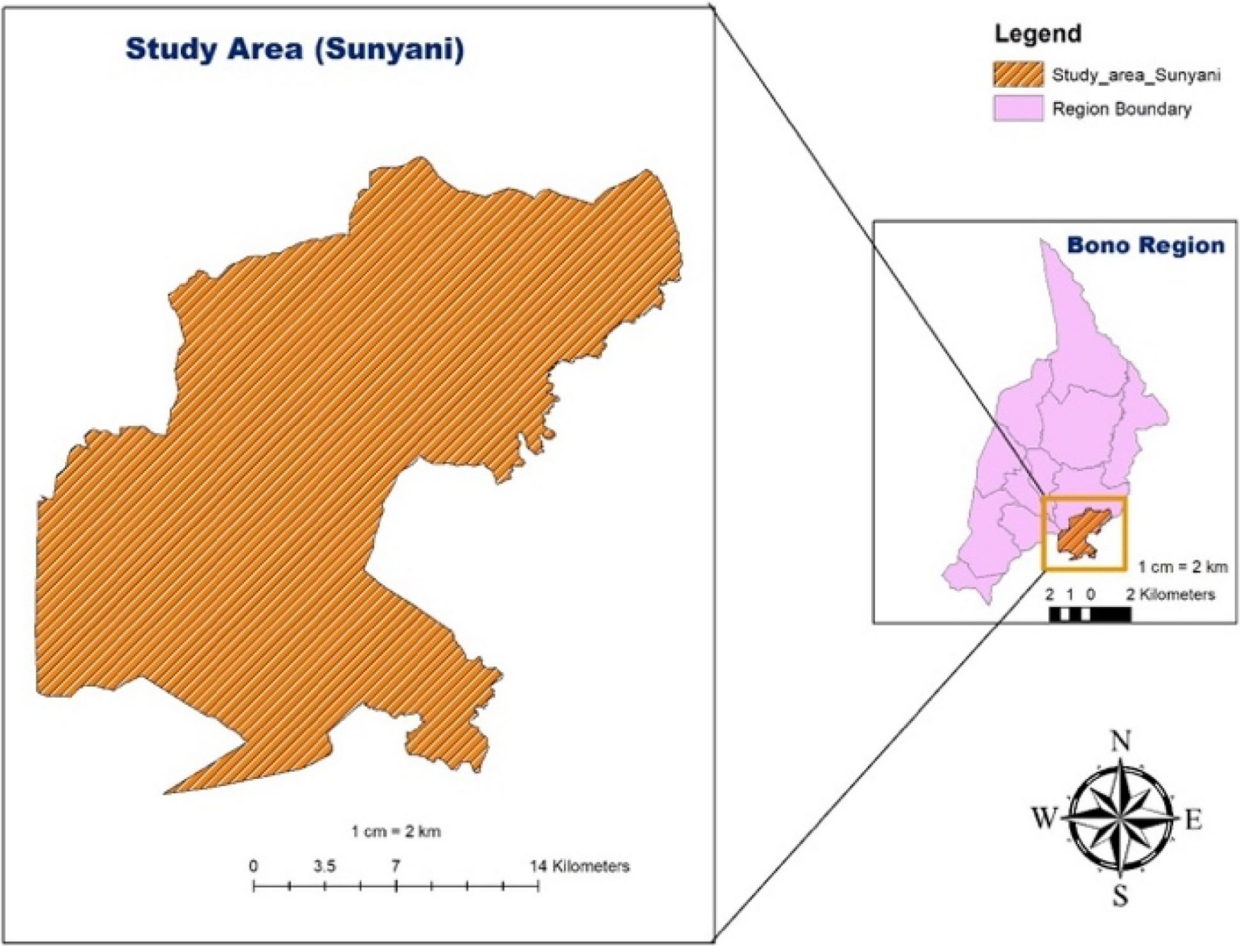
Map of Sunyani Municipal in the Bono region

It has Sunyani as its municipal capital and now Bono Regional Capital as shown in (Figs. 1 and 2).

The current district population as estimated by the 2020 housing and population census is 134,958. The total population of the Heath Administration Districts and sub-districts in the Municipality is 193,595 with women of childbearing age contributing 27.0% (52,271) of the total population. The fertility rate is 3.6 births/woman in 2021. The expected Pregnancies/Deliveries is7744 per annum. The Maternal Mortality ratio for 2021 was reported at 84/100,000 live births.

The municipality has 35 health facilities which include regional hospitals, municipal hospitals, health centers, school clinics, private clinics, quasi-facilities, private maternity homes, Christian Health Association of Ghana (CHAG) facilities, and 34 functional CHPS zones (3 compounds) in six sub-municipalities. All these categories offer either basic or comprehensive emergency obstetric care.

The regional hospitals provide a wide range of specialized medical services and advanced diagnostic and therapeutic procedures and serve as referral centers for smaller hospitals in the region. On the other hand, the municipal hospitals are medium-sized and offer a broad spectrum of medical services including general surgery, obstetrics and gynecology, Pediatrics, and internal medicine and serve a local municipal population, and refer more complex cases to the regional hospitals. The District Health Information Management System (DHIMS) is a comprehensive platform used for collecting, managing, and analyzing health data at various levels of the healthcare system in Ghana. Health facilities at all levels collect data on various health indicators using standardized forms and electronic devices which is entered into the DHIMS either directly at the facility level or through the district Health offices. It uses an online platform that allows for real-time data entry and access. It contains tools for data validation and supports customized dashboards for data analysis and reporting.

### Study population

A checklist was developed using the WHO MDSR Guidelines, CDC updated Guidelines, and the IDSR 3rd Edition guidelines for the evaluation. This checklist was used to interview various stakeholders, including disease control officers, health information officers, physician assistants, midwives, community-based volunteers, reproductive health nurses in public, private, and faith-based organizations, as well as the district director at the Municipal Health Directorate.

### Sampling procedure

We purposively selected eleven (11) health facilities out of thirty-five based on their capacity to provide comprehensive maternal and child health services and their history of reporting maternal deaths over the past five years. The evaluation only selected facilities that reliably reported maternal deaths. Four (4) hospitals, including one (1) regional, one (1) municipal, one (1) private, and one (1) faith-based. The selection also considered the diversity of hospital settings. Similarly, five (5) government-operated health facilities from the sub-district were chosen due to their inclusion in a reporting platform and their provision of basic emergency obstetric and neonatal care services. This choice aimed to capture diversity in healthcare settings. Additionally, two (2) Community Health Planning and Services (CHPs) compounds were included in the sample.

The selection of interviewees from different hospitals prioritized midwives, surveillance officers, and health information officers. A minimum of three individuals were interviewed at each facility to cover specifically, individuals who were on duty at their workstations on the day of the interview. This method aimed to capture perspectives from essential personnel directly engaged in maternal health services and surveillance within the selected facilities.

### Standard operations of the Maternal Death Surveillance and Response System in Sunyani Municipal

**Fig. 3:**
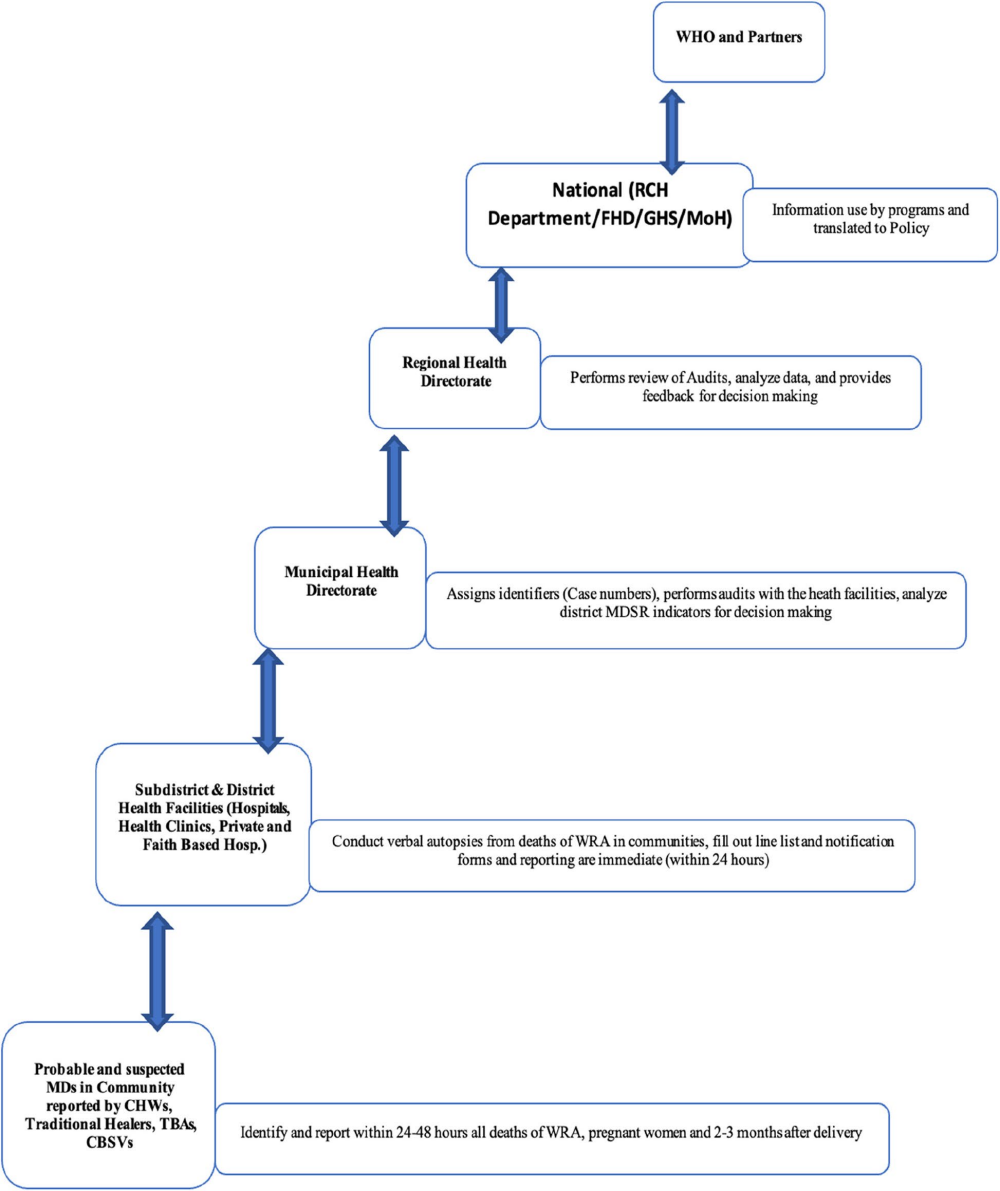
Information flow chart in the maternal death surveillance system

The Maternal Death Surveillance and Response System (MDSR) in Sunyani Municipal operates through a continuous cycle comprising four key components: identification and notification, death review or audits, analysis and recommendations, and response and monitoring. Maternal death information is reported by Community-Based Volunteers, Traditional Birth Attendants, Traditional Leaders, and Religious Leaders, either directly or through phone calls or SMS to health workers. Health facilities also detect and record maternal deaths within their premises. Both community and facility-based notifications undergo comprehensive audits, including qualitative investigations into medical causes, contributing factors, and avoidability. The audit teams, which typically include doctors, nurses, midwives, and other relevant healthcare personnel, review the cases to identify gaps or issues in the care provided. Additionally, district and regional health authorities oversee the audit process to ensure consistency and adherence to national guidelines. The audit results are coded, entered into a database, and used to make recommendations for preventing future deaths (Fig. 3).

### Data collection

Quantitative data were collected through a thorough records review. The primary source was the electronic dataset on Maternal Deaths from 2017 to 2022, which included performance indicators exported to MS Excel spreadsheets from the District Health Information Management System (DHIMS2) platform. Monthly data were cross-verified for accuracy by comparing them with records from facilities’ consulting room registers and tally sheets.

### Sources for data collection

- Midwife Returns Form: A summary form compiling aggregated data from the maternity register, covering metrics like the number of deliveries, antenatal visits, and maternal outcomes. This form is essential for service delivery monitoring and trend identification.
- Line Listing Form: Used for recording and tracking individual cases of maternal deaths, with detailed information on age, parity, cause and place of death, and contributing factors. This form is crucial for identifying maternal mortality patterns.
- IDSR Report Forms: Tools for reporting maternal deaths as part of the Integrated Disease Surveillance and Response strategy.
- Case-Based Notification Forms: Provide specific information for the notification of individual cases.
- Case Notes: Offer comprehensive clinical details about each maternal death.
- Maternal Death Audit Reports: Summarize findings from investigations and audits of maternal deaths.
- Community Registers: Local documentation of maternal health and outcomes.
- Rumor Logbook: Captures unverified reports of maternal deaths from community sources, supplementing formal records and enhancing system comprehensiveness through follow-up investigations.

### Data collection and validation

- Semi-structured interviews were conducted using an interview guide aligned with CDC’s guidelines for evaluating surveillance systems. Data were gathered on knowledge of case definitions, data collection, analysis, and dissemination practices, system integration, and resource availability.
- Participants were briefed on the study’s objectives and confidentiality, and their verbal consent was obtained. The interviews were conducted in English by the Principal Investigator. The interviews lasted 24 to 70 min (average 35 min) and were audio-recorded and transcribed verbatim.

### Data analysis

Quantitative analysis: The variables analyzed descriptively (median, interquartile range, frequencies, proportions/ percentages) included sociodemographic variables and the respondents’ attributes that were assessed using Microsoft Excel 2021 and Epi Info version 7.

Qualitative analysis: Direct content analysis was employed to identify patterns and themes within the qualitative data. A coding scheme was developed after reviewing five randomly selected transcripts and was then applied deductively to the rest of the transcripts. Coding was performed manually using Microsoft Word. The themes that emerged were carefully reviewed, defined, and documented in a codebook to ensure the analysis’s rigor [9, 10]. This codebook contained definitions for each code and guidelines on when to apply them, clarifying how certain statements should be categorized. The codebook was used to help systematically organize data into themes and sub-themes. This ensured that each participant’s unique experiences were considered while maintaining consistency during coding.

### Ethical consideration

The Director of the Diseases Surveillance Department of the Ghana Health Service approved the access and use of the data for this review. Permission was also officially sought from the Bono Regional Director of Health Services and Sunyani Municipal Health Directorate for the use of the data. Written consent was obtained from health workers before interviews were conducted. To ensure confidentiality, coded respondent identification numbers were used in place of names, and aggregate analyses were done on data. Data held on computers were encrypted with a password which was made available only on a need-to-know basis, without any form of coercion or undue influence.

## Results

### Overview of respondents

Thirty-seven (37) respondents were interviewed from 11 health facilities. About 51.4% (19/37) of respondents were females. The median age of respondents was 33 years, with IQR of 28–36 yrs Midwives consisted the highest proportion of respondents and 94.6% (35/37) worked at government-run health facilities or institutions as shown below (see Table 1).

**Table 1:** Showing demographic characteristics of respondents in Sunyani East Municipal, 2022

Demographic characteristics	Frequency (*n* = 37)	Percentage
***Age (yrs.)***		
18–24	2	5.4
25–34	12	32.4
35–44	17	45.9
45–54	5	13.5
55 +	1	2.7
***Sex***		
Male	19	51.4
Female	18	48.6
***Cadre***		
Medical doctors	4	10.8
Midwives	12	32.4
Disease surveillance officers	8	21.6
Health information officers	8	21.6
Public Health physicians	4	10.8
Community based volunteers	1	2.7
***Health facility type***		
Regional Hospital	2	5.4
Municipal Hospital	7	18.9
Private Hospital	8	21.6
Health centers	15	40.5
CHPs	5	13.5

### The usefulness of the surveillance system

Of the respondents interviewed, 54.1% (20/37) had recorded maternal deaths at their facilities. All facilities had functional maternal death review committees, and all reported deaths at the facilities were audited. Additionally, the audits were conducted within the health facilities, as there was no evidence of verbal autopsies being performed. Most of the respondents 86.5% (32/37) were able to explain the trend of maternal deaths, with all hospital facilities having a display of summaries and charts of maternal death trends and other obstetric indicators over the 5-years. About 75.7% (28/37) were able to explain how the surveillance data has been used for public health action to avoid preventable maternal deaths at their facility.

Many respondents expressed that analysis and use of data from maternal death audits and reviews have been instrumental in reducing preventable maternal deaths. A nurse from the municipal directorate revealed how the data successfully prompted initiatives to enhance awareness and engage communities in efforts to decrease maternal mortality within both the district and the broader region.

*“When we realised*,* the deaths were increasing*,* we introduced the zero maternal death campaign between 2019 and 2020 and that helped us greatly to forge collaborations and improve awareness amongst stakeholders and community members”Reproductive Health Nurse*,* 2022*.

It was also a common opinion that the recommendations from the reviews are periodically analyzed, and interventions are tailored accordingly, as highlighted by a doctor at the Obstetrics and Gynecology unit, Regional Hospital in Bono.

*“In 2019*,* we found that a significant proportion of maternal deaths 60% (9/15) were due to indirect (non-obstetric) causes. In response*,* we enhanced our collaboration with other departments to provide comprehensive care and improved critical care management for patient care. This significantly reduced our overall deaths the following year " Medical doctor*,* 2022*.

To ensure more deaths are preventable, action points from various reviews are meticulously followed up to avoid recurrence. The Health Information Officer described the effectiveness of following up on action points, responses, and recommendations.

*“We developed an action points tracker to regularly monitor the implementation of recommendations. Additionally*,* we assessed the proportion of action points achieved to evaluate the impact of the audits and reviews.” Health Information Officer*,* 2022*.

### The simplicity of the system

Following the interview, 54.1% (20/37) respondents had completed a Maternal Death Case Notification form. Even though case definitions were not translated to local languages, 85% (17/20) reported the forms were precise and easy to use. All those who had completed the maternal death notification forms knew the reporting channels. Only 15% (3/20) felt that training was required on understanding some variables as well as the reporting channels.

### The flexibility of the system

Majority (86.7%, 32/37) of the respondents reported that the case definition, which is a standard has not changed over the years. However, there has been a significant challenge in reporting maternal deaths and other health-related events from communities, primarily due to the decreased motivation among community-based volunteers who play a crucial role in linking communities with health centers and providing essential services. One of the heath staff at the sub-district shared this view;

*“When the community-based volunteers were well motivated and their work was incentivized*,* we used to receive information on all deaths from the communities but this rarely happen nowadays” -Midwife*,* 2022*.

### Acceptability of the system

Most of the participants 94.6% (35/37) were willing to participate or notify a maternal death from their facilities. Additionally, 94.6% (35/37) were willing to be involved in the audits and felt it was more of a duty to attend audit meetings, even though only about 5% (2/37) feared that the deaths will be blamed on their actions or inactions.

### Stability of the system

Many 94.6% (35/37) reported that there were no inconsistencies in reporting maternal deaths, especially from health facilities and there were no interruptions from lack of resources or a change in procedures. However, the majority 86.5% (32/37) said that the ill-motivated and ineffective community-based volunteers have affected the detection and reporting of maternal deaths in the communities. The MDSR data was accessible mainly through the DHMIS in almost all facilities. Lack of communication equipment to receive, store, transfer, or secure data was reported in most facilities 72.7% ( 8/11).

### Timeliness and completeness of the system

Of the forms reviewed at the Municipal and the Regional directorate levels, 80% (20/25) were reported after 24 h although all the facilities eventually report on all maternal deaths. Additionally, 88% (22/25) were investigated within seven days. The timeline for implementation of preventive measures varied with the issues identified as some were for immediate action and others needed more planning and resources as well as support from the Regional or National Level. Immediate feedback was given during the audit reviews for appropriate action at the various levels.

### Data quality

Most facilities did not have a line list and the paper-based maternal death notification forms were incomplete or entirely not available. Maternal death line list was only available at the regional and municipal health directorates. About 68.0% (17/25) of the forms reviewed from the hospitals were filled out completely. Data from the line list was in sync with the data reported in the DHIM 2 except for the data from the regional hospital with some variations as only Direct Maternal Deaths were reported in the DHIMs, (Table 2).

**Table 2:** Differences in data from source document and that entered in the DHIMS for Direct and Indirect Maternal Death in the Brong Ahafo Regional Hospital, 2017–2021

Year	Total	Indirect	Direct	Entered in DHIMS	% Difference
**2017**	20	8	12	12	40.0
**2018**	17	8	9	9	47.1
**2019**	17	9	6	6	52.9
**2020**	3	1	1	1	33.3
**2021**	14	7	7	6	50.0

All the hospitals visited had health information officers who periodically analyzed the data. However, data analysis at the Sub-district and CHPS levels was very limited, with only about 50% (10 out of 20) analyzing maternal care indicators at these facilities.

### Representativeness of the system

Data were reported by age, hospital, and community and included all populations. All maternal deaths were recorded and classified into Direct or Indirect based on the causes. However, all deaths reported for the study period were institutional deaths with no records of community deaths reported.

## Discussions

The evaluation aimed to assess the maternal death surveillance system’s usefulness, its surveillance attributes, and its effectiveness in meeting its objectives. The findings reveal that the maternal death surveillance system in the Sunyani East Municipality was useful, simple, flexible, and acceptable, meeting several of its intended objectives. However, several challenges persist, including the unavailability of reporting tools at the subdistrict level, poor data quality and management, insufficient human resources, particularly community-based volunteers, weak information sharing and linkages with communities, and issues with implementing recommendations. These findings indicate that while the system has strengths, there are significant areas that require improvement to enhance its overall effectiveness.

The Maternal Death Surveillance System was found to be useful, mirroring a study conducted in Hwange District, Zimbabwe, which similarly characterized the system as beneficial [11]. These findings resonate with global best practices, emphasizing the importance of maternal death surveillance systems in effectively reducing preventable maternal deaths [12].

The system demonstrates simplicity, aligning with findings from an evaluation conducted in the Mutare District of Zimbabwe [13]. The positive response from healthcare professionals regarding the ease of using Maternal Death Case Notification forms speaks to the user-friendly nature of the system. This ease of use likely contributes to efficient data collection and reporting, facilitating smoother integration of the surveillance system into routine healthcare practices.

Our evaluation revealed that the system is acceptable, similar to findings from a study in Zimbabwe by Maphosa et al. [11]. The high willingness of healthcare professionals to participate in the surveillance system and audits suggests a positive reception and acceptance of the system within healthcare facilities. Studies conducted in Zimbabwe’s Mutare District also highlighted a similar pattern of willingness to report [13]. Despite some concerns about potential blame, the overwhelming willingness indicates a sense of duty among professionals to actively engage in maternal death reporting and audits. This study aligns with existing literature emphasizing the importance of fostering a no-name, no-blame principle to encourage comprehensive reporting in maternal death reviews [14]. Conversely, research from Guinea highlighted how underreporting was driven by fear of blame or punishment [15]. Similar findings emerged from Benin, where fear was cited as a significant barrier to reporting or participating in MDSR processes [16]. Additionally, Kouanda et al. noted that concerns over potential repercussions contributed to underreporting [17]. Tura et al. [18] also discussed the political sensitivities linked to maternal death reporting, where higher-level officials and political leaders sought to minimize the occurrence of reported maternal deaths, thus contributing to underreporting.

The system encounters challenges related to data quality. Issues such as incomplete or unavailable paper-based maternal death notification forms raise concerns about the reliability of information at the facility level. Mutsigiri-Murewanhema et al. reported similar experiences with shortages of notification forms at the facility [13]. This issue is likely exacerbated by staff who have never reported maternal deaths and may overlook the importance of having these forms readily available. The absence of maternal death notification forms at subdistrict-level facilities results in delays in reporting and affects data accuracy. This issue is critical in ensuring proper classification of cases and reducing morbidity and mortality [12]. Variations between the line list and DHIMS further emphasize the need for standardized reporting practices and regular data quality assessments. Similar observations were made in a study emphasizing the need for robust health information systems and adequate funding for maintaining data quality [19]. Additionally, poor data quality was observed in a Burkina Fao study related to poor record-keeping and documentation [20]. The findings emphasize that reporting maternal deaths from health facilities demonstrates a stable component of the MDSR system. The findings reveal that while the standard case definition has remained consistent over time, significant challenges persist, particularly with the availability of devices and storage equipment for maternal health data, especially at the municipal and sub-district levels. Stable MDSR systems typically sustain consistency through well-established communication networks and adequate logistical support, including essential equipment for data collection, storage, transmission, and analysis. Strategic investments in infrastructure and capacity-building efforts are vital for strengthening these systems [21]. However, in rural and low-resource environments, these issues are amplified by resource limitations, complicating the ability to achieve consistent and reliable community-level reporting.

The reports indicate that timeliness is prioritized in the MDSR system in Sunyani. However, challenges persist, especially in the comprehensive implementation of preventive measures that require additional resources or support from higher levels. Timely reporting and response are critical to reducing maternal mortality, as delays in data reporting and analysis can impede prompt interventions. Research, such as that by Whiting-Collins et al. highlights that systems with structured review timelines and local accountability mechanisms tend to achieve more effective outcomes [22]. Nevertheless, achieving both completeness and timeliness often depends on the availability of consistent resources and adequate staff training—areas where Sunyani’s system, similar to those in many LMICs, may experience significant limitations. The system exhibited a degree of flexibility, demonstrated by the adoption of new data collection tools and the integration of electronic forms for immediate death notifications within the weekly IDSR framework. However, phone-based notifications and extensive reliance on paper-based reporting remained significant. The ability to revise objectives, update monitoring and evaluation indicators, and align maternal mortality definitions with ICD-10 standards underscored the system’s adaptability while maintaining operational efficiency. These adjustments highlight an inherent capacity to adapt, despite persistent challenges in fully transitioning to digital reporting. Such findings align with those reported by Krimi et al. [23], which emphasize the adaptability of systems facing similar logistical and resource-related challenges.

The system was partially representative. Although the data’s comprehensive reporting by age, hospital, and community showcases the system’s ability to capture a wide range of information, the absence of records for community deaths highlights a potential limitation, questioning the system’s representativeness beyond institutional settings. The findings also show that all deaths reported at the institutional level were from labor ward records, which could lead to an underestimation of deaths due to a lack of comprehensive notification systems. These findings align with a study in Rwanda, which identified a similar challenge in the pattern of notification for maternal and perinatal deaths [14].

There were variations in the implementation of preventive measures following audits and maternal death reviews. The underreporting of community-based maternal deaths is a well-acknowledged phenomenon. To address this issue, some countries, such as South Africa, have made the notification of all pregnancy-related deaths mandatory by law [24]. According to Smith et al. [19], maternal deaths are prone to underestimation due to incomplete data and misclassification, particularly in cases occurring at home or within specific age cohorts. Mutsigiri-Murewanhema et al. [13] similarly found that health workers primarily report deaths at the health facility due to the difficulty in verifying deaths at the community level. Maternal deaths that occur in the community often go unreviewed, and consequently, community-related recommendations are not followed up on [24]. This may also be due to the lack of formal feedback or follow-up mechanisms following the review [17]. In contrast, Bandali et al. [25] demonstrated a review of the Maternal Death Review in Nigeria, showing that despite reporting challenges at the subnational level, there were concrete efforts focused on the use of MDR findings, accountability, and follow-ups. The confirmation of maternal death is predominantly feasible in hospital settings, as these events are exclusively certified by medical doctors, posing challenges in verification at the subdistrict level. The underreporting issue is further explicable in light of the complexities associated with establishing and sustaining robust reporting mechanisms at the community level. The identification and variation in reported deaths among health facilities were crucial findings, echoing the results of a study by Yameogo et al. in Burkina Faso [20].

Another contributing factor to the underreporting of community-based maternal deaths is the lack of training and motivation among community-based volunteers and stakeholders to report suspected maternal deaths. This observation concurs with the guidelines outlined by the World Health Organization (WHO) [12], which underscore the impediments linked to community-based reporting. It’s important to consider that the lack of locally translated case definitions may not have significantly impacted facility reporting due to the level of medical education. However, this is a crucial factor for community reporting and could affect the accuracy of data from the community [26]. Moreover, a study conducted in South Africa by Moodley et al. [27] corroborates these findings, underscoring the imperative of not solely focusing on facility-based maternal death notifications but also fostering connections with communities to incentivize reporting. It is also important to consider challenges to reporting from the communities, including inadequate Civil Registration and Vital Statistics systems. This may sometimes lead to reliance on ad hoc surveys and facility-based systems, which often lack national representativeness and lead to significant underestimation of maternal deaths.

The evaluation reveals a gap in information sharing between health workers and communities. It also demonstrated that feedback sharing with the community was limited, despite some problems having root causes related to the communities. A study in Rwanda also noted a similar finding in which dissemination of audit findings was not implemented in all health facilities [14]. This disconnect impedes efforts to address factors contributing to maternal deaths at both facility and community levels, emphasizing the importance of effective communication and collaboration [28].

While evaluating the maternal death surveillance system in Sunyani Municipal, Bono region, our study encountered certain limitations. The selection of facilities was based on their ability to provide either basic or comprehensive maternal and child health services, which may introduce potential bias by excluding locations where deaths might occur due to facility or provider limitations. The primary mode of information collection relied on subjective assessments, although we augmented our findings with evidence obtained from facility records, including reports and minutes. Unfortunately, due to the unavailability of data on specific system attributes, we were unable to comprehensively examine all aspects of the system. Nevertheless, despite these challenges, we objectively assessed key attributes, contributing valuable insights into the performance of the maternal death surveillance and response system in Sunyani Municipal, Bono Region.

## Conclusions

The maternal death surveillance system is useful and meets most of its objectives. It was found to be useful, acceptable, and flexible. However, data incompleteness and unavailability were identified, as well as the underrepresentation of community deaths. Staff needs to be trained on how to correctly fill in case-based forms and generate a facility-based line list, and there is a need to strengthen the training and motivation of community-based volunteers on community case searches. Mentorship on the correct filling of case-based forms was conducted during the evaluation, and findings and recommendations were communicated to the Municipal Health Directorate for action. Given the small sample size, the recommendations may need further assessment for their appropriateness and feasibility before generalizing them to other districts. These findings and lessons learned may apply to other settings implementing MDSR systems, especially those facing similar resource and reporting challenges, suggesting a potential for broader contextual adaptation.

## Supplementary Information

Supplementary Material 1.

Supplementary Material 2.

## Data Availability

The datasets generated and/or analyzed during the current study are available from the corresponding author upon reasonable request. Restrictions may apply to the availability of some data due to privacy or ethical restrictions.
