# Supplementary material for: Evaluating maternal death surveillance and response system in Sunyani Municipality of Bono region in Ghana from 2017-2021
Source: BMC Health Serv Res. 2024 Dec 18;24:1578. doi: 10.1186/s12913-024-12023-7 (PMC11653697; doi:10.1186/s12913-024-12023-7)
Supplement: Supplementary file 1 — Supplementary Material 1. [file 12913_2024_12023_MOESM1_ESM.docx]

**CHECKLIST FOR EVALUATING THE ATTRIBUTES OTH THE MTAERNAL DETH SURVEILLANCE SYSYTEM**

**DEMOGRAPHIC CHARACTERISTICS**

Region ____________ District ______________

Total population ______ Urban ________ Rural _________ Male ________ Female ____________

Respondent’s Name______________________________________________________

Cell phone no _____________ Title/Qualification ______________________

**USEFULNESS (see how many objectives are met)**

1. Is the surveillance system able to estimate the incidence of maternal death and show *the trend of maternal deaths? Yes  No*

*Comments________________________________________________________________________________________________________________________________________*

1. *Is the system able to indicate major causes of maternal deaths? Yes  No*

*Comments________________________________________________________________________________________________________________________________________*

1. *Are there preventive measures applied to the data and recommendations from MDSR to decrease the death of mothers? Yes  No*

*Comments________________________________________________________________________________________________________________________________________*

1. *Can the Data from MDSR be used for decision making or can it stimulate research? Yes  No*

*Comments________________________________________________________________________________________________________________________________________*

1. *Should the system remain in place? Yes  No*

*Comments________________________________________________________________________________________________________________________________________*

**SIMPLICITY**

CDC defines the simplicity of a public health surveillance system as both its structure and ease of operation while still meeting its objectives.

1. *Is it easy to use the case definition of MDSR by all health workers? Yes  No*

*Comments________________________________________________________________________________________________________________________________________*

1. *Are Data Collection/Case notification forms clear and precise? Yes  No*

*Comments________________________________________________________________________________________________________________________________________*

1. *What is the average time taken to complete a maternal death notification form? Yes  No*

*Comments________________________________________________________________________________________________________________________________________*

1. *Are Reporting channels are known/clear for those involved? Yes  No*

*Comments________________________________________________________________________________________________________________________________________*

1. *Is training required to work in the MDSR Surveillance System (or can be done by orientation)? Yes  No*

*Comments________________________________________________________________________________________________________________________________________*

**FLEXIBILITY**

Flexibility is the ability of the system to adapt to changing information needs and operating conditions with minimal additional cost.

1. *Has there been any change in the case definition of MDSR in the past years? Yes  No*

*Comments_________________________________________________________________________________________________________________________________­_*

1. *Was it difficult to adapt (involved more time and personnel) to the new case definition of MDSR Yes  No*

*Comments_________________________________________________________________________________________________________________________________­_*

1. *Has there been a change in the reporting system or is a new technology added? Yes  No*

*Comments_________________________________________________________________________________________________________________________________­_*

1. *Has there been a change in the funding for MDSR activities? Yes  No*

*Comments_________________________________________________________________________________________________________________________________­_______*

1. *Is it integrated with the HMIS and IDSR? Yes  No*

*Comments_________________________________________________________________________________________________________________________________­_­­­______*

**ACCEPTABILITY**

Acceptability is the willingness of persons and organizations to participate in a surveillance system, including those who operate the system, report cases of the disease, or use the data

1. *Are they willing to continue participating in the MDSR? Yes  No*

*Comments_________________________________________________________________________________________________________________________________­_­­_____*

1. *Is there a refusal in completing the questionnaire? Yes  No*

*Comments_________________________________________________________________________________________________________________________________­______*

1. *Are they willing to notify a maternal death? Yes  No*

*Comments_________________________________________________________________________________________________________________________________­______*

1. *Are they Willing to participate in maternal death review meetings? Yes  No*

*Comments_________________________________________________________________________________________________________________________________­______*

1. *Comfortable when assigned to participate in verbal autopsy activities? Yes  No*

*Comments_________________________________________________________________________________________________________________________________­______*

1. *Is the Community willing to participate. Yes  No*

*Comments_________________________________________________________________________________________________________________________________­______*

1. *Participation Rate of the facility or respondents Yes  No*

*Comments_________________________________________________________________________________________________________________________________­______*

*In addition, completeness and timeliness will also be assessed as a proxy of acceptability*

**STABILITY**

is the ability of a surveillance system to collect, manage, and provide data without failure and to be operational when needed. Stability is the reliability and availability of the surveillance system. Reliability is the ability to collect, manage and provide data properly without failure. Availability is the ability of a surveillance system to be operational when it is needed.

1. *Has the system been interrupted due to a lack of resources for the past year Yes  No*

*Comments_________________________________________________________________________________________________________________________________­______*

1. *What has affected the operations (procedures and activities) of the MDSR Yes  No*

*Comments_________________________________________________________________________________________________________________________________­______*

1. *Has there been consistency in reporting? Yes  No*

*Comments_________________________________________________________________________________________________________________________________­______*

1. *Is the MDSR data was ready for use when needed for planning and other
   purposes? Yes  No*

*Comments_________________________________________________________________________________________________________________________________­______*

1. *Staff available to run the surveillance system Yes  No*

*Comments_________________________________________________________________________________________________________________________________­______*

1. *Is there Available communication equipment to receive, edit, store, transfer, and secure data and other needed resources for the surveillance system? Yes  No*

*Comments_________________________________________________________________________________________________________________________________­______*

**SENSITIVITY**

The sensitivity of a surveillance system can be assessed by the proportion of cases detected by the surveillance system and the ability of the system to monitor changes in the number of cases over time

1. *Does the case definition able to pick all cases? Yes  No*

*Comments_________________________________________________________________________________________________________________________________­______*

1. *What were the total numbers of suspected cases of the condition under surveillance? __*
2. *Can you verify this number from that in the register? ____*
3. *How many Suspected maternal deaths were not maternal deaths after MDR? ____*
4. *How many maternal deaths were missed (Difference between the no of notifications and the total maternal deaths registered) ______*

**DATA QUALITY**

The quality of data is influenced by the clarity of surveillance forms, the quality of training and the supervision of persons who complete the maternal death notification forms and the amount of care that is exercised in managing the surveillance data.

1. *What training has personnel in charge of data received? ________________________*

*_______________________________________________________________________*

1. *Is the collected Data Consistent with Maternal Death Yes  No*

*Comments_________________________________________________________________________________________________________________________________­______*

1. *How many trained-on Data Quality___________*
2. *How many forms are filled properly? _________*
3. *How many forms are not correctly filled? _______*
4. *Is the data analysed Yes  No If yes, How is it analysed? _________*

**TIMELINESS**

Timeliness refers to the speed at which data is transmitted between different levels in the surveillance system.

1. *How many cases of maternal deaths were reported ____*
2. *How many facility deaths were reported within 24 hrs ______*
3. *How many Community Deaths were reported within 48 hrs _____*
4. *When was the investigation done? ________*
5. *When was the review done ________*
6. *When was the control and preventive measures implemented ______*
7. *Within which time frame was the feedback given? ______*

**REPRESENTATIVENESS**

Is the extent to which the system accurately describes the occurrence of the disease over time and its distribution in the population by place and person

1. *Is the surveillance system enabled to follow all cases and events in the entire communities? Yes No*

*Comments_________________________________________________________________________________________________________________________________­______*

1. *Are data reported by age? Yes No*

*Comments_________________________________________________________________________________________________________________________________­______*

1. *Are data reported by communities in the district? Yes No*

*Comments_________________________________________________________________________________________________________________________________­______*

1. *Are data reported by all health facilities in the district? Yes No*

*Comments_________________________________________________________________________________________________________________________________­______*

1. *Are data from special populations like clients of sex workers and migrants captured? Yes No*

*Comments_________________________________________________________________________________________________________________________________­______*
