# Supplementary material for: Evaluating maternal death surveillance and response system in Sunyani Municipality of Bono region in Ghana from 2017-2021
Source: BMC Health Serv Res. 2024 Dec 18;24:1578. doi: 10.1186/s12913-024-12023-7 (PMC11653697; doi:10.1186/s12913-024-12023-7)
Supplement: Supplementary file 2 — Supplementary Material 2. [file 12913_2024_12023_MOESM2_ESM.docx]

## ***Case Definitions Used***

**Standard case definition**: The 10^th^ International Classification of Diseases (ICD-10) defines Maternal death as “the death of a woman while pregnant or within 42 days of the delivery or termination of the pregnancy, irrespective of the duration and site of the pregnancy, from any cause related to or aggravated by the pregnancy or its management but not from accidental or incidental causes”.

**Suspected case definition**: Is defined as the death of any woman while pregnant or within 42 days of the termination of pregnancy.

**Probable maternal death**: Deaths among women of reproductive age (WRA), not clearly due to incidental or accidental causes.
